# Chloroform in Hypochondriasis

**Published:** 1854-02

**Authors:** 


Chloroform in Hypochondriasis.—At the meeting of the College of Physicians in Ireland in June, Professor Osborne stated that he had lately, in two cases, opportunities of observing a peculiar effect of chloroform taken into the stomach, in controlling the depressing and saddening feelings belonging to hypochondriasis. Considering that state to be produced by a depraved sensibility of the stomach or colon, and frequently of both, he was led to the internal employment of chloroform, which, being promptly volatilized at the temperature of the stomach and before being decomposed by the process of digestion, ought to be expected to act as a local anæsthetic, even though the dose should not be sufficient to produce any change in the function of the brain.—*Dub. Quar. Journal.*

